# Hypervirulent *Klebsiella pneumoniae* is emerging as an increasingly prevalent *K. pneumoniae* pathotype responsible for nosocomial and healthcare-associated infections in Beijing, China

**DOI:** 10.1080/21505594.2020.1809322

**Published:** 2020-09-12

**Authors:** Chao Liu, Pengcheng Du, Nan Xiao, Fansen Ji, Thomas A. Russo, Jun Guo

**Affiliations:** a Department of Infectious Diseases, Peking University Third Hospital, Beijing, China; b Institute of Infectious Diseases, Beijing Ditan Hospital, Capital Medical University, and Beijing Key Laboratory of Emerging Infectious Diseases, Beijing, China; c Department of Clinical Laboratory, Beijing Tsinghua Changgung Hospital, School of Clinical Medicine, Tsinghua University, Beijing, China; d Department of Basic Medical Sciences, School of Medicine, Tsinghua University, Beijing, China; e Department of Medicine, University at Buffalo, State University of New York, Buffalo, NY, USA; f Veterans Administration Western New York Healthcare System, Buffalo, New York, USA; g Department of Pulmonary and Critical Care Medicine, Beijing Tsinghua Changgung Hospital, School of Clinical Medicine, Tsinghua University, Beijing, China

**Keywords:** Hypervirulent *Klebsiella pneumoniae*, multi drug resistance, carbapenemase, epidemiology, nosocomial infection, clinical characteristics, risk factor

## Abstract

**Objectives:**

Hypervirulent Klebsiella pneumoniae(hvKp) is an increasingly important pathogen. Tracking its epidemiology and evolving antimicrobial resistance will facilitate care.

**Methods:**

A retrospective study was conducted in two hospitals. We collected the clinical data. Antimicrobial and virulence-associated phenotype and genotype, sequence type, and whole genome sequencing of selected strains were performed. HvKp was defined by the presence of some combination of _p_rmpA, _p_rmpA2, iucA, iroB, and peg-344, genes shown to accurately identify hvKp.

**Results:**

Of 158 Kp clinical isolates, 79 (50%) were hvKp. Interestingly, 53/79 (67.1%) of hvKp strains were isolated from patients with nosocomial infection and 19/79 (24.1%) from patients with healthcare-associated infection, but only 7/79 (8.8%) from patients with community-acquired infections. Importantly, 27/53 (50.9%) and 4/19 (21.1%) of hvKp nosocomial and healthcare-associated isolates, respectively, were multi-drug-resistant (MDR); 25/53 (47.2%) and 5/19 (26.3%) expressed ESBLs and 14/53 (26.4%) and 2/19 (10.5%) were carbapenem-resistant. Of the hvKp isolates from community-acquired infection, 0/7 (0%) were MDR and 0/7 (0%) were carbapenem-resistant. Additionally, unique characteristics of nosocomial, healthcare-associated, and community-acquired hvKp infection were identified. In summary, 50% of K. pneumoniae infections were caused by hvKp. A concerning, novel finding from this report is a major shift in hvKp epidemiology. Ninety-one percent of hvKp infections were nosocomial or healthcare-associated, and 43.1% of these isolates were MDR.

**Conclusions:**

These data suggest that hvKp may be replacing classical K. pneumoniae as the dominant nosocomial and healthcare-associated pathotype. Ongoing surveillance is needed to determine if this trend is occurring elsewhere.

## Introduction

*Klebsiella pneumoniae* (Kp) is a clinically important Gram-negative bacterium [1], especially in China [2]. Kp has evolved into two distinct pathotypes: hypervirulent *Klebsiella pneumoniae* (hvKp) and classical *Klebsiella pneumoniae* (cKp) [3,4]. cKp has been called a superbug based on its acquisition of various antimicrobial resistance genes and is primarily responsible for healthcare-associated infections [5]. HvKp is more virulent than cKp and was initially described to cause community-acquired infections [6]. Early studies attributed a hypermucoviscous phenotype, (defined by a positive string test) as the defining trait for hvKp strains. However, not all hvKp strains are hypermucoviscous and some cKp strains possess this characteristic [3,7–9]; thus, the string test was an imprecise way to identify hvKp when used alone. This has created some confusion in the literature. Recently, multiple biomarkers-including *peg-344, iroB, iucA, _p_rmpA*, and *_p_rmpA2* that are most often present on the hvKp virulence plasmid or in some instances some of these (*iro, peg-344, rmpA*) can be located on integrative and conjugative elements (ICE) [10], have been shown to have a high diagnostic accuracy for identifying hvKp [9]. Data using these markers would be predicted to generate more reliable data on various aspects of hvKp infection.

Until recently, in most previous studies the majority of hvKp were antimicrobial sensitive, in contrast to cKp strains, which were more frequently multi- and extensively drug-resistant (MDR, XDR) [6]. Unfortunately, MDR hvKp, extended-spectrum-β-lactamase (ESBL) producing hvKp, polymyxin resistant hvKp, and carbapenem-resistant hvKp is emerging, especially in China [11–14]. Of concern was the observation that the transfer of a KPC-producing plasmid into an hvKp isolate was not associated with fitness cost [15]. Even more disturbing was a fatal outbreak that occurred in China due to an XDR cKp strain acquiring the hvKp virulence plasmid [16]. These reports emphasize the importance of ongoing surveillance of hvKp infection and antimicrobial resistance trends.

Thus, to further investigate the clinical characteristics, risk factors, prevalence and antibiotic resistance of hvKp, a retrospective study in two hospitals in Beijing using newly validated biomarkers for definition hvKp (the presence of some combination of *peg-344, iroB, iucA, _p_rmpA*, or *_p_rmpA2*) was conducted in Beijing. Surprisingly, an epidemiologic shift may be occurring for hvKp. Nosocomial and healthcare-associated infections accounted for >90% of hvKp infections and hvKp is challenging cKp as the dominant nosocomial and healthcare-associated *K. pneumoniae* pathotype. Further, 43.1% of nosocomial and healthcare-associated isolates were MDR or XDR, whereas the community-acquired strains remained relatively antimicrobial susceptible. These findings are concerning and have significant implications for patient care and outcomes.

## Materials and methods

### Enrolled patients

A retrospective study was conducted on 159 *Kp* culture-positive patients at the one hospital (n = 84) and the other hospital (n = 75) in Beijing (Hospital A and B) from 2008 to 2018. Information was obtained from electronic medical records, including basic demographic characteristics, underlying diseases, antimicrobial agent exposure, the site of infection, and use of invasive devices. Thirty-day mortality was recorded. Sequential organ failure assessment (SOFA) score and Charlson comorbidity index (CCI) were calculated. The main inclusion criteria included: 1) age >18 years old; 2) Kp was identified in ≥2 specimens cultured, was the dominant species in the culture, or was cultured from a sterile site; 3) symptomatic infection from the site where Kp was cultured. Exclusion criteria included: 1) insufficient clinical data; 2) bacterial strain not viable after storage; 3) duplicate isolates from the same patient within 3 months.

For this study, nosocomial infections were defined as patients who developed infection after being admitted for more than 48 hours. Healthcare-associated infection was defined as an infection in a patient who had prior interaction with the healthcare system before developing their infection (e.g. recent surgery within 90 days, dialysis, lines/catheters in place, reside in a long-term care facility, hospital discharge within the last 90 days and others), and community-acquired infection was defined as cases without any prior exposure to healthcare facilities. Metastatic infection was defined as more than one site of infection due to the infecting Kp strain, excluding the bloodstream, as described [17].

The protocol for this study was approved by the Beijing Tsinghua Changgung Hospital Ethics Committee (18,116-0-01), and the Guidelines for Human Experimentation (PRC) were followed throughout. Informed consent was not obtained for this study since clinical data were de-identified.

### *Clinical* Kp *strains*

All strains were stored at −80°C. Strains were initially identified by Vitek compact 2 system and confirmed by the MALDI-TOF mass spectrometry. HvKp were defined by the presence of some combination of *peg-344, iroB, iucA, _p_rmpA*, or *_p_rmpA2* [9]. Hypermucoviscous phenotype was defined by a positive string test as described [18].

### Antimicrobial susceptibility testing and phenotypic detection of ESBLs

Antimicrobial susceptibility testing was performed by Vitek 2. The antimicrobials tested included imipenem, meropenem, amikacin, gentamicin, tobramycin, aztreonam, cefazolin, cefepime, ceftriaxone, ceftazidime, ciprofloxacin, levofloxacin, ampicillin/sulbactam, piperacillin/tazobactam, and trimethoprim/sulfamethoxazole. Antimicrobial susceptibility testing results were interpreted using 2017 Clinical and Laboratory Standards Institute (CLSI) guideline breakpoints. ESBL was also detected by the Vitek 2. MDR was defined as resistance to three or more different antimicrobial classes and XDR was defined as susceptible to only one or two classes [19].

### Detection of virulence-associated genes

Genomic DNA of all *Kp* strains were extracted for use in polymerase chain reaction (PCR) assays. Capsular type-specific (*cps*) gene (*K1, K2, K5, K20, K54*, and *K57*) and *_p_rmpA, _p_rmpA2, iucA, iroB, peg-344, peg-589* were identified as described [7,9]. The primers utilized for these studies are listed in Supplementary Table 1.

### Multilocus Sequence Typing (MLST) for Kp strains

MLST was performed according to the protocol described on the website (*http://bigsdb.pasteur.fr/klebsiella/klebsiella.html*) using seven housekeeping genes (*gapA, mdh, phoE, tonB, infB, pgi, and rpoB*) (Supplementary Table 1). Allelic profiling and sequence types (STs) were also determined by the use of this website.

### Whole genome sequencing and analysis for ST11 and ST23 strains

To determine if infections due to ST11 and ST23 strains were sporadic or represented an outbreak, whole genome sequencing (WGS) was conducted. ST11 (n = 38) and ST23 (n = 11) strains were chosen for WGS because they were the most prevalent ST_S_ identified. Briefly, the whole genomic DNA was extracted and sequenced using Illumina sequencing method on the NovaSeq platform by constructing paired-end (PE) library (NEB dsDNA Fragmentase) with average insertion lengths of 150 bp. The clean data were obtained using fastQC and assembled using SPAdes v3.13 by the default parameters [20]. The virulence genes and plasmids were identified by sequence comparison using BLAST v2.2.18 [21].

The complete genome sequence of *K. pneumoniae* HS11286 (accession no.: NC_016845) and SGH-10 (accession no.: CP025080) was used as the reference to perform phylogenetic analysis. The sequencing read was mapped to the reference using bowtie 2 v2.2.8 and the single nucleotide polymorphisms (SNPs) were identified by using Samtools v1.9 and combined together according to the reference using the iSNV-calling pipeline we constructed previously [22,23]. The high-quality SNPs supported by more than 5 reads of mapping quality >20 remained. The recombination sites were detected by Gubbins [24] and the concatenated sequences of filtered polymorphic sites conserved in all strains (core genome SNPs, cgSNPs) were used to perform phylogenetic analysis using maximum likelihood method by FastTree [25]

### Statistical analysis

Data were analyzed using the SPSS software (version 20.0). Data were analyzed as means ± standard deviation (SD). Continuous variables were assessed by Student’s *t*-tests. For categorical variables, x^2^ or Fisher’s exact test was used for group comparisons. Logistic regression analyses were conducted for risk factors and independent risk factor (the variables with *P* < 0.05 were included) was performed by multivariable logistic regression analysis. All tests were 2-tailed. A *P*-value<0.05 was considered to be statistically significant. No multiple-testing corrections have been applied.

### Data availability

All genome sequences have been deposited in the NCBI Sequencing Read Archive database under the accession number SRP224044 (Supplementary Table 2).

## Results

### Patient characteristics, clinical syndromes, and infecting pathotype

One-hundred and fifty-eight *K. pneumoniae* infection cases from two different hospitals were enrolled in this study. One hundred and thirty-three (83.6%) were males and most patients were older than 65 years (the mean age: 78.18 ± 15.16 y). The majority of cases (74.1%, 117/158) were nosocomial infections and 47 (29.7%, 47/158) cases admitted into the ICU. Thirty-one cases (19.6%,31/158) were healthcare-associated and only 10 cases (6.3%, 10/158) were community acquired. (Table 1)

**Table 1.** Clinical characteristics of hvKp infection.

	Community-acquired infection (*n* = 10)		Healthcare-associated infection (*n* = 31)		Nosocomial infection (*n* = 117)		
Clinical characteristic	HvKp(7)	cKp(3)	HvKp(19)	cKp(12)	HvKp(53)	cKp(64)	*P value^※^*
Basic demographics							
Age	75.29 ± 9.12	76.00 ± 22.52	76.84 ± 17.95	80.67 ± 11.55	78.21 ± 18.09	78.52 ± 12.65	0.914
**Metastatic infection**	2(28.6%)	0(0%)	8(42.1%)	0(0%)	**21(39.6%)**	**14(21.9%)**	**0.037**
Male	5(71.4%)	2(66.7%)	18(94.7%)	11(91.7%)	45(84.9%)	52(81.3%)	0.601
Underlying diseases							
Pulmonary disease	5(71.4%)	2(66.7%)	16(84.2%)	9(75.0%)	30(56.6%)	47(73.4%)	0.056
Diabetes	5(71.4%)	2(66.7%)	11(57.9%)	7(58.3%)	25(47.2%)	25(39.1%)	0.378
Cardiovascular disease	5(71.4%)	1(33.3%)	10(52.6%)	5(41.7%)	38(71.7%)	43(67.2%)	0.599
**Cerebrovascular disease**	1(14.3%)	0(0%)	7(36.8%)	6(50.0%)	**35(66.0%)**	**28(43.8%)**	**0.016**
Cancer	1(14.3%)	0(0%)	5(26.3%)	5(41.7%)	12(22.6%)	21(32.8%)	0.224
Surgery within 3 months	0(0%)	0(0%)	3(15.8%)	6(50.0%)	17(32.1%)	24(37.5%)	0.590
Digestive disease	3(42.9%)	0(0%)	7(36.8%)	5(41.7%)	30(56.6%)	35(54.7%)	0.836
Urinary disease	2(28.6%)	1(33.3%)	3(15.8%)	4(33.3%)	21(39.6%)	26(40.6%)	0.912
Usage of Glucocorticoid within 7 days	1(14.3%)	0(0%)	1(5.2%)	0(0%)	16(30.2%)	10(15.6%)	0.059
Antibiotics exposure within 90 d	0(0%)	0(0%)	9(47.4%)	4(33.3%)	49(92.5%)	61(95.3%)	0.516
Immunosuppression	1(14.3%)	0(0%)	5(26.3%)	5(41.7%)	21(39.6%)	30(46.9%)	0.431
Catheter							
Central intravenous catheter	0(0%)	0(0%)	8(42.1%)	0(0%)	42(79.2%)	42(65.6%)	0.103
**Urinary catheter**	0(0%)	0(0%)	7(36.8%)	5(41.7%)	**48(90.6%)**	**49(76.6%)**	**0.045**
Endotracheal tube	0(0%)	0(0%)	3(15.8%)	0(0%)	34(64.2%)	30(46.9%)	0.062
**Gastrostomy tube**	0(0%)	0(0%)	8(42.1%)	7(58.3%)	**48(90.9%)**	**48(75.0%)**	**0.029**
Drainage tube	0(0%)	0(0%)	1(5.2%)	4(33.3%)	19(35.8%)	22(34.4%)	0.868
Infection type							
Wound	1(14.3%)	0(0%)	0(0%)	1(8.3%)	2(3.8%)	5(7.8%)	0.599
Pneumonia	5(71.4%)	2(66.7%)	13(57.9%)	10(83.3%)	42(79.2%)	49(76.6%)	0.728
Urinary infection	1(14.3%)	1(33.3%)	8(21.1%)	1(8.3%)	17(32.1%)	17(26.6%)	0.513
Bacteremia	0(0%)	0(0%)	0(0%)	0(0%)	6(11.3%)	8(12.5%)	0.845
Liver abscess	0(0%)	0(0%)	1(5.2%)	0(0%)	1(1.9%)	2(3.1%)	1.000
Other abscess	1(14.3%)	0(0%)	5(26.3%)	0(0%)	3(5.7%)	1(1.6%)	0.225
Abdominal infection	1(14.3%)	0(0%)	0(0%)	0(0%)	7(13.2%)	7(10.9%)	0.706
**Sepsis**	2(28.6%)	0(0%)	10(52.6%)	4(33.3%)	**43(81.1%)**	**38(59.4%)**	**0.011**
WBC	13.29 ± 10.72	9.27 ± 2.05	14.55 ± 11.76	9.03 ± 3.68	9.87 ± 3.77	11.00 ± 5.25	0.192
NEU%	68.00 ± 15.56	68.47 ± 3.88	73.76 ± 19.67	78.08 ± 9.58	77.22 ± 10.06	77.73 ± 11.30	0.779
TP	72.14 ± 6.34	64.33 ± 4.51	63.63 ± 14.12	66.27 ± 5.06	63.86 ± 6.39	64.26 ± 5.49	0.712
ALB	40.29 ± 3.25	37.33 ± 1.53	34.53 ± 9.38	34.74 ± 4.19	33.19 ± 3.62	33.33 ± 4.39	0.852
CCI>4	2(28.6%)	0(0%)	8(42.1%)	8(66.7%)	29(54.7%)	39(60.9%)	0.497
**SOFA>6**	2(28.6%)	0(0%)	11(57.9%)	5(41.7%)	**35(66.0%)**	**26(40.6%)**	**0.006**
Admitted in the ICU^#^	1(14.3%)^#^	0(0%)	4(21.1%)^#^	1(8.3%)	15(28.3%)	26(40.6%)	0.164
30 day mortality *	1(14.3%)	0(0%)	1(5.2%)	3(25.0%)	8(15.1%)	13(20.3%)	0.464

The P value <0.05 was shown in the bold values. TP: Total protein; ALB: Albumin; WBC: White blood cell count; NEU%: Neutrophils percentage; CCI: Charlson comorbidity index; SOFA: Sequential organ failure assessment.*: Death or life-sustaining therapy withheld due to poor prognosis; #: patient with community-acquired infection or healthcare-associated infection was admitted or transferred to the ICU. *^※^*: statistical tests were performed only for the nosocomial group.

In these 158 cases, 45 patients had multiple sites of infection, resulting in 218 infectious syndromes. The most common was pneumonia (n = 121, 55.5%; 60 (49.6%) due to hvKp), followed by urinary tract infection (n = 45, 20.6%; 26 (57.8%) due to hvKp), abdominal infection (n = 15, 6.9%; 8 (53.3%) due to hvKp), abscess (n = 14, 6.4%; 11 (78.6%) due to hvKp), bacteremia (n = 14, 6.4%; 6 (42.9%) due to hvKp) and wound infection (n = 9,4.1%; 3 (33.3%) due to hvKp). Sepsis developed in 97/158 patients (61.4%; 55 (56.7%) due to hvKp) and the overall 30-day mortality was 16.5% (n = 26; 10 (38.5%) due to hvKp); in 5 of these patients life-sustaining support measures were withheld due to poor prognosis (Table 1).

Sixty-one distinct STs and fifteen new STs were identified among the 158 *Kp strai*ns. ST11 (n = 38; 24.1%; 18/38 were hvKp) was the most prevalent ST identified in this study, followed by ST23 (n = 11; 7.0%; 11/11 were hvKp), ST17 (n = 6; 3.8%; 2/6 were hvKp), ST111 (n = 5;3.2%; 4/5 were hvKp). These STs accounted for 38.0% (60/158) of the total strains. ST23 (11/11), ST35 (3/3), ST65 (3/3), and ST218 (3/3) were only detected in hvKp isolates.

**Figure 1.**
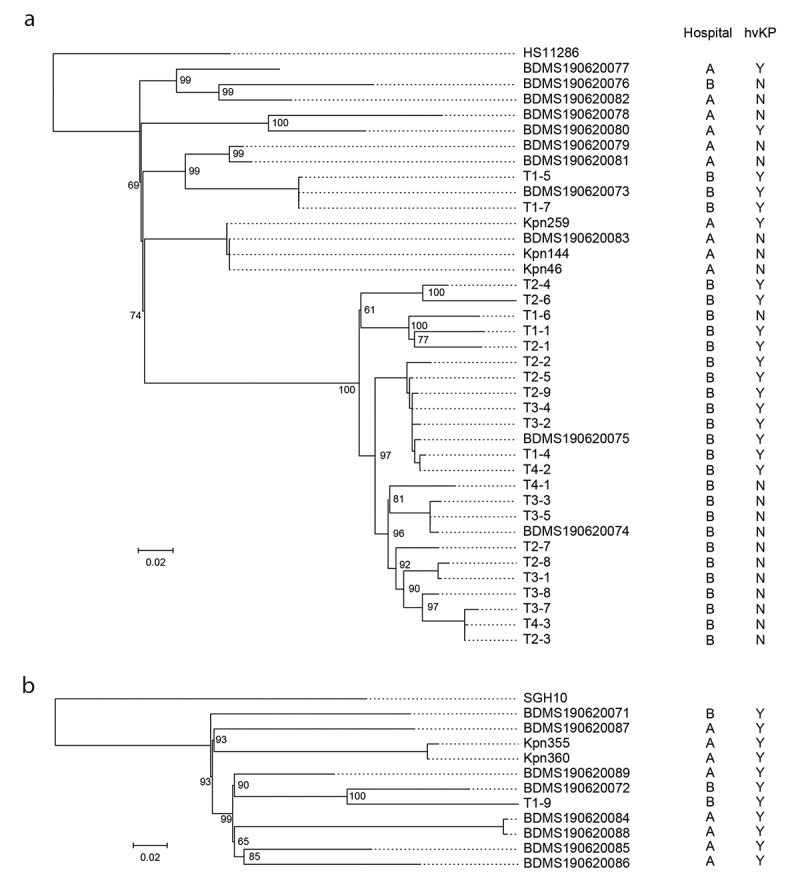
The phylogenetic tree of ST11 and ST23 strains isolated in this study.

We performed genome sequencing and phylogenetic analysis to study the relationships and identify the potential nosocomial transmission of the 38 ST11 and 11 ST23 isolates obtained in this study. The sequencing depth of the 49 isolates was 257.9 ± 72.4 in average. By comparison with the genome of HS11286, 869 cgSNPs were identified in ST11 strains, accounting for 108 ± 59 pairwise SNPs in average (range: 1–234). By comparison with the genome of SGH10, 1,125 cgSNPs were identified in ST23 strains, accounting for 239 ± 60 pairwise SNPs in average (range: 4–333). The phylogenetic tree was constructed based on these cgSNPs (Figure 1). Meanwhile, multiple lineages emerged in both STs (Figure 1), and the numbers of cgSNPs among different lineages were high (Supplementary Table 3). These results demonstrated that most of the ST11 and ST23 strains we obtained were not clonal, and therefore represented sporadic infections or from different transmission events or small outbreaks.

ST11 is a common XDR-ST in China that was initially only described in cKp strains. However, recently, it appears that ST11 has acquired either a portion of an hvKp virulence plasmid that contained *iucA* and *rmpA2* (but not *iroB, rmpA*, or *peg-344*) [16] or the complete pLVPK-like virulence plasmid [26,27] thereby conferring a hypervirulent phenotype. Mounting evidence supports that both sublineages are disseminating in China [26,28,29]. Of the 38 ST11 strains reported here, 18/38 were hvKp; 11/18 possessed *iucA* and *rmpA2* (but not *iroB, rmpA*, or *peg-344*) and WGS data demonstrated that these eleven strains harboured a pVir-CR-hvKp4-like plasmid as was previously reported [16]. These data are consistent with spread of this strain within China. One of the eighteen ST11 hvKp strains possessed the pLVPK-like virulence plasmid. Lastly, 9/18 of these ST11 hvKp strains were XDR. Of the 18 hvKp ST11 strains, 18/18 caused healthcare-associated or nosocomial infection, respectively. Since WGS demonstrated that the ST11 hvKp strains belonged to different lineages and were not clonal, the introduction of the majority of these hvKp strains into the healthcare setting would appear to be occurring by multiple independent events and did not represent limited introduction followed by clonal spread (i.e. an outbreak). However, once introduced, these isolates could also spread within hospital. Two highly related clusters were found among ST11-hvKp. One cluster contained eight strains (SNP<25). Three cases were from ICU and the four strains isolated from Internal Medicine Department. Another cluster included three strains, which were distributed in ICU and Rehabilitation department. These strains could represent spread of hvKp within the hospital.

ST23 is a ST that almost always is seen in hvKp strains that cause community-acquired infection. Of the 11 ST23 strains, 9/11 caused healthcare-associated or nosocomial infection. Since WGS demonstrated that none of these 11 hvKp ST23 strains were clonal, the introduction of these hvKp strains into the healthcare setting would also appear to be occurring by multiple independent events and did not represent an outbreak.

Overall, half of 158 (79/158) isolates were hvKp and 61/79 (77.2%) and 4/79 (5.1%) of hvKp and cKp strains were hypermucoviscous (hmvKp) respectively. Thirty-eight of fifty-three (71.7%) hvKp nosocomial isolates were hmvKp, while 3/64 (4.7%) of cKp strains demonstrated this phenotype (*P* < 0.001). Capsule types *K1*(17.0% vs 0%; *P* = 0.001), *K2* (13.2% vs 1.6%; *P* = 0.034) and *peg-589* (58.5% vs 0%; *P* < 0.001) were significantly associated with the hvKp strain cohort when compared to the cKp strain cohort.(Table 2)

**Table 2.** Virulence phenotype and genotype of hvKp.

	Community-acquired infection (*n* = 10)		Healthcare-associated infection (*n* = 31)		Nosocomial infection (*n* = 117)		
Virulence	HvKp(7)	cKp(3)	HvKp(19)	cKp(12)	HvKp(53)	cKp(64)	*P value ^※^*
K serotype							
***K1***	0(0%)	0(0%)	7(36.8%)	0(0%)	**9(17.0%)**	**0 (0%)**	**0.001**
***K2***	1(14.3%)	0(0%)	1(5.2%)	0(0%)	**7(13.2%)**	**1(1.6%)**	**0.034**
*K5*	0(0%)	0(0%)	0(0%)	0(0%)	0(0%)	0(0%)	
*K20*	1(14.3%)	0(0%)	0(0%)	0(0%)	1(1.9%)	1(1.6%)	1.000
*K54*	1(14.3%)	0(0%)	0(0%)	1(8.3%)	0(0%)	1(1.6%)	1.000
*K57*	1(14.3%)	1(33.3%)	1(5.2%)	1(8.3%)	3(5.7%)	1(1.6%)	0.482
***rmpA***	5(71.4%)	0(0%)	11(57.9%)	0(0%)	**32(60.4%)**	**0(0%)**	*#*
***rmpA2***	5(71.4%)	0(0%)	12(63.2%)	0(0%)	**41(77.4%)**	**0(0%)**	*#*
*crmpA*	2(28.6%)	0(0%)	1(5.2%)	0(0%)	2(3.8%)	0(0%)	0.203
**Hypermucoviscosity**	7(100.0%)	0(0%)	16(84.2%)	1(8.3%)	**38(71.7%)**	**3(4.7%)**	**0.000**
***iucA***	5(71.4%)	0(0%)	14(73.7%)	0(0%)	**43(81.1%)**	**0(0%)**	*#*
***iroB***	6(85.7%)	0(0%)	15(78.9%)	0(0%)	**22(41.5%)**	**0(0%)**	*#*
***peg-344***	6(85.7%)	0(0%)	14(73.7%)	0(0%)	**22(41.5%)**	**0(0%)**	*#*
***peg-589***	4(57.1%)	0(0%)	13(68.4%)	0(0%)	**31(58.5%)**	**0(0%)**	**0.000**
***rmpA+rmpA2+ iucA+iroB+peg-344***	4(57.1%)	0(0%)	8(42.1%)	0(0%)	**12(22.6%)**	**0 (0%)**	**0.000**
***iucA+rmpA2^&^***	1(14.3%)	0(0%)	1(5.3%)	0(0%)	**9(17.0%)**	**0 (0%)**	**0.000**
pLVPK-like*	1(14.3%)	0(0%)	5(26.3%)	0(0%)	6(11.3%)	**0 (0%)**	*
pVir-CR-HvKP4-like*	0(0%)	0(0%)	3(15.8%)	0(0%)	8(15.1%)	**0 (0%)**	*

The P value <0.05 was shown in the bold values. &: Strains that only have these two genes among *iucA, rmpA, rmpA2, iroB, peg-344*. *: The pLVPK-like and pVir-CR-HvKP4-like plasmids were achieved by the ST11 and ST23 strains. Due to the potential gaps of Next-generation Sequencing, statistical analysis for plasmids was not conducted. ^#^: HvKp are defined on the basis of possession of one or more of these markers (the combination of *iroB, iucA, rmpA/A2* and *peg-344*). Therefore, it doesn’t make sense to test for differences of hvKp vs cKp. *^※^*:statistical tests were performed only for the nosocomial group.

### Prevalence and risk factors of hvKp compared to cKp in nosocomial infection

Detailed characteristics and outcomes of nosocomial hvKp compared to cKp infection are shown in Table 1. One hundred seventeen Kp isolates (117/158,74.1%) caused a nosocomial infection, of which 53 (45.3%) and 64 (54.7%) were due to hvKp and cKp respectively.

Univariate analysis did not demonstrate significant differences in infectious syndromes between the hvKp and cKp groups. By contrast, compared with the cKp group, more patients with hvKp infection had cerebrovascular disease (66.0% versus 43.8%; *P* = 0.016) as their underlying disease, metastatic infection was more common (39.6% versus 21.9%; *P* = 0.037), as was sepsis (81.1% versus 59.4%; *P* = 0.011) and a SOFA score >6 (66.0% vs 40.6%, *P* = 0.006), but there was no difference in 30-day mortality. Further, hvKp infected patients were more likely to have an indwelling urinary catheter (90.6% versus 76.6%; *P* = 0.045) and a gastrostomy tube (90.9% versus 75.0%; *P* = 0.029). (Table 1)

On multivariate analysis, cerebrovascular disease (OR = 2.415) and SOFA>6 (OR = 2.304) remained as independent variables associated with hvKp compared to cKp infection (Table 3).

**Table 3.** Risk factors for nosocomial hvKp infection.

Variable	Univariate OR (95% CI)	*P*-value	Multivariate OR (95% CI)	*P*-value
Metastatic infection	2.344(1.044–5.262)	0.039		
Cerebrovascular disease	2.500(1.177–5.309)	0.017	2.415(1.072–5.437)	0.033
Gastrostomy tube	3.200(1.086–9.432)	0.035		
SOFA>6	2.842(1.334–6.054)	0.007	2.304(1.029–5.158)	0.042

### Prevalence of hvKp compared to cKp in healthcare-associated and community-acquired infection

Detailed characteristics and outcomes of healthcare-associated and community-acquired hvKp infection compared to cKp infection are shown in Table 1. Thirty-one Kp isolates (31/158,19.6%) caused a healthcare-associated infection, of which 19 (61.3%) and 12 (38.7%) were due to hvKp and cKp respectively. Ten Kp isolates (10/158, 6.3%) caused community-acquired infection, of which 7 (70%) and 3 (30%) were due to hvKp and cKp respectively. Univariate analysis was not conducted due to the small sample size. However, metastatic infection (28.6% versus 0%), abscess (14.3% versus 0%), SOFA score >6 (28.6% versus 0%), and 30-day mortality (14.3% versus 0%) occurred more commonly in the hvKp infected patients compared to those infected with cKp.(Table 1)

### Antimicrobial resistance in hvKp compared to cKp isolates

Overall the antimicrobial resistance rate for cKp strains was still greater than rates for hvKp (Table 4). However, the difference in resistance rates between cKp and hvKp was less than expected. Also, as predicted, resistance rates were low for both cKp and hvKp in the community-acquired infection group, higher in the healthcare-associated infection group, and highest in the nosocomial infection cohort. Surprisingly in the nosocomial group, 27/53 (50.9%) of hvKp were MDR or XDR, 25/53 (47.2%) expressed ESBLs, and 14/53 (26.4%) were carbapenem-resistant. Nonetheless, cKp isolates from nosocomial infections were more likely to be MDR than hvKp strains (68.8% versus 50.9%, *P* = 0.05) and were significantly more likely to possess an ESBL (67.2% versus 47.2%, *P* = 0.029). Likewise, compared to hvKp strains, cKp isolates were significantly more resistant to gentamicin (45.3% versus 30.2%, *P* = 0.038), cefazolin (75% versus 50.9%, *P* = 0.007), ceftriaxone (65.6 versus 47.2%, *P* = 0.045), and trimethoprim/sulfamethoxazole (53.1% versus 26.4%, *P* = 0.003). Surprisingly, resistance to carbapenems was similar for cKp and hvKp strains. Due to small numbers, statistical analysis was not conducted in resistant rates of hvKp and cKp isolated from community-acquired and healthcare-associated infections (Table 4).

**Table 4.** Antibiotics resistance patterns of hvKp.

	Community-acquired infection (*n* = 10)		Healthcare-associated infection (*n* = 31)		Nosocomial infection (*n* = 117)		
Antibiotic agent	hvKp(7)	cKp(3)	hvKp(19)	cKp(12)	hvKp(53)	cKp(64)	*P-*value^※^
**MDR**	0(0%)	0(0%)	4(21.1%)	6(50.0%)	**27(50.9%)**	**44(68.8%)**	**0.050**
**ESBLs** Amikacin **Gentamicin** Ampicillin/Sulbactam Aztreonam **Cefazolin** Cefotetan Cefepime **Ceftriaxone** Ceftazidime Ciprofloxacin Levofloxacin **Trimethoprim/Sulfamethoxazole** Piperacillin/Tazobactam Imipenem Meropenem Tobramycin	1(14.3%) 0(0%) 0(0%) 2(28.6%) 0(0%) 1(14.3%) 0(0%) 0(0%) 1(14.3%) 0(0%) 0(0%) 0(0%) 0(0%) 0(0%) 0(0%) 0(0%) 0(0%)	1(33.3%) 0(0%) 0(0%) 0(0%) 0(0%) 1(33.3%) 0(0%) 0(0%) 0(0%) 0(0%) 0(0%) 0(0%) 0(0%) 0(0%) 0(0%) 0(0%) 0(0%)	5(26.3%) 0(0%) 2(10.5%) 6(31.6%) 4(21.1%) 5(26.3%) 3(15.8%) 2(10.5%) 5(26.3%) 4(21.1%) 4(21.1%) 4(21.1%) 3(15.8%) 2(10.5%) 2(10.5%) 2(10.5%) 2(10.5%)	4(33.3%) 0(0%) 1(8.3%) 5(41.7%) 2(16.7%) 6(50.0%) 1(8.3%) 2(16.7%) 5(41.7%) 1(8.3%) 4(33.3%) 4(33.3%) 5(41.7%) 1(8.3%) 1(8.3%) 1(8.3%) 3(25.0%)	**25(47.2%)** 6(11.3%) **16(30.2%)** 31(58.5%) 23(43.4%) **27(50.9%)** 15(28.3%) 22(41.5%) **25(47.2%)** 23(43.4%) 25(47.2%) 24(45.3%) **14(26.4%)** 15(28.3%) 13(24.5%) 14(26.4%) 16(30.2%)	**43(67.2%)** 13(20.3%) **29(45.3%)** 47(73.4%) 29(45.3%) **48(75.0%)** 17(26.6%) 21(32.8%) **42(65.6%)** 29(45.3%) 32(50.0%) 29(45.3%) **34(53.1%)** 23(35.9%) 18(28.1%) 19(29.7%) 25(39.1%)	**0.029** 0.189 **0.038** 0.088 0.094 **0.007** 0.834 0.331 **0.045** 0.836 0.760 0.997 **0.003** 0.380 0.661 0.695 0.317

The P value <0.05 was shown in the bold values. *^※^*:statistical tests were performed only for the nosocomial group.

## Discussion

One of the challenges in studies on hvKp has been the lack of a test to differentiate cKp from hvKp strains. Although this posed less of a problem with community-acquired infections that presented with a classical clinical syndrome, it has proved problematic for healthcare-associated and nosocomial infections. A strength of this study was that strains were defined as being cKp or hvKp based on the presence of *iucA, rmpA, rmpA2, iroB*, and *peg-344*, biomarkers that were recently shown to be highly accurate for differentiating hvKp from cKp strains [9]. Further, cases assessed were from two hospitals in Beijing, China. Several important findings were identified from this study. First, of the 158 *K. pneumoniae* strains, 79 (50%) were hvKp isolates. Second, and most importantly, the 91% (72/79) of the hvKp strains were isolated from patients with healthcare-associated (n = 19) or nosocomial infection (n = 53). These data support the concept that either an increasing number of healthcare-associated and nosocomial infections are due to hvKp or these infections were previously unrecognized due to the inability to differentiate hvKp versus cKp infections in these settings. Regardless, an important conclusion from this study is that hvKp is not just a cause of community-acquired infections but also healthcare-associated and nosocomial infections, at least in Beijing, China. If the incidence of hvKp nosocomial infections is truly increasing, one might conclude that hvKp may be replacing cKp as the dominant nosocomial pathotype. If this is the case, this will prove to be extremely problematic. The management of hvKp infection could differ from cKp infection (e.g. surveillance for occult infection, source control, site-specific antimicrobial therapy); hence, an awareness that hvKp should be considered for healthcare-associated and nosocomial *K. pneumonia* infections and its identification would be important for patient care [4]. Further, some community-based studies have demonstrated that pneumonia due to hvKp has increased mortality compared to *S. pneumoniae* [30]; given the increased virulence of hvKp it is reasonable to speculate that may result in increased mortality in this vulnerable population compared to cKp in the healthcare setting.

Although this study demonstrated that patients with nosocomial hvKp infection had a significantly higher SOFA score, and were more likely to have sepsis, there was not a difference in 30-day mortality. Whether this was due to an insufficient number of cases or perhaps 30-day mortality does not reflect attributable mortality awaits future studies. The report by Gu et al., however, clearly demonstrated the potentially devastating effects of hvKp infection in patients in an ICU [16].

There have been several reports that have shown the hvKp strains are no longer exclusively acquired in the community, but are being increasingly recognized within health-care systems. Gu et al. reported on a lethal ICU outbreak due to hvKp [16]. A more recent report by Harada et al. demonstrated that more than half of hvKp infections from seven different hospitals in Japan were healthcare-associated or nosocomial [31]. Additionally, Chih-Han Juan, et al. reported that hvKp were prevalent in community-acquired, healthcare-associated and hospital-acquired pneumonia patients [32]. These data are consistent with our findings that hvKp appears to be infiltrating into our healthcare systems.

Another important observation was the relatively high level of antimicrobial resistance observed in nosocomial hvKp isolates; 27/53 (50.9%) were MDR or XDR, 25/53 (47.2%) expressed ESBLs, and 14/53 (26.4%) were carbapenem-resistant. Such strains are more challenging to treat and outcomes are worse [14,16,33–35]. Further, pending susceptibilities, choosing an empirical regimen that is active will be more difficult. A previous study demonstrated that the detection rate of ESBL-hvKp was 12.6%, less than what was observed in our study [36]. Likewise, in a multicenter study focused on the elderly infected with *K. pneumoniae*, 26% were ESBL-producing hvKp and 11.5% of hvKp isolates were carbapenem-resistant [37]. If this trend continues, this new breed of XDR-hvKp threatens the viability of current therapeutic approaches and control of nosocomial infection [16].

Increased antimicrobial resistance in hvKp strains in China, in part, is due to ST11 Kp, a predominant clone in China. ST11 was previously identified as cKp and its reduced susceptibility to most available antibiotics was mainly due to the acquisition of KPC-2 [36,38,39]. From 2012 to 2016, an epidemiological survey of carbapenem-resistant *Enterobacteriaceae* conducted in China indicated that the rapid rise in the detection rates of MDR/XDR strains had been attributed to ST11 clone [39,40], which is a common cause of hospital infection outbreaks [41,42]. One mechanism that could explain increasing antimicrobial resistance in hvKp strains from China is the acquisition of the pLVPK-like virulence plasmid (pVir-CR-hvKP4) by ST11 strains endowing it with a hvKp hypervirulent phenotype [16]. In our study, nine nosocomial hvKp strains possessed the biomarker profile of being positive for *iucA* and *rmpA*2, but negative for *iroB, rmpA*, and *peg-344*. Of these strains, 10/11 were ST11, consistent with the hvKp ST11 strains described by Gu et al. [16]. However, confirmation awaits more detailed sequence analysis.

In fact, our data suggest that the apparent changing epidemiology of hvKp, in part, is the result of existing cKp, ST11, acquiring the virulence-like plasmid replacing cKp among nosocomial infections. ST11 strains that possess both antimicrobial-resistant determinants and increased virulence would be predicted to have an increased ability to survive and cause infection in the healthcare environment. In addition, hvKp strains that were initially associated with community-acquired infection (e.g. ST23) are now also responsible for healthcare-associated or nosocomial infection as was seen with 9/11 ST23 strains in this study.

In our study, one community-acquired hvKp strain possessed the biomarker profile of being positive for *iucA* and *rmpA*2, but negative for *iroB, rmpA*, and *peg-344* consistent with the hvKp ST11 strains described by Gu et al. [16]. Albeit this is only a single strain, it suggests the possibility that nosocomial hvKp strains may be spreading into the community.

An incompletely studied area is whether all strains defined as having some combination of *iucA, rmpA, rmpA2, iroB*, and *peg-344* are equally virulent. In the study that defined these markers as highly predictive of hvKp, nearly all strains possessed all five of these markers, which is not surprising since they are usually linked on the hvKp virulence plasmid [9]. However, in our study some strains possess only some of these markers. It would be important and interesting to see if such strains were equally virulent in an infection model. In the Gu study, the ST11 hvKp strain SH-1 harbored the pLVPK-like virulence plasmid pVir-CR-hvKP4 (178,154 bp) [16]. However, pVir-CR-hvKP4 had 41,231 bp deletion compared to pLVPK, which included the virulence genes *rmpA, iro, and peg-344*; the *iuc* genes and *rmpA*2 were retained and the presence of *rmpA2* appeared to confer a hypermucoviscous phenotype. This strain was virulent in the *Galleria mellonella* model, but was not assessed in a mouse model. However, recently published data have shown that the *Galleria mellonella* model does not accurately differentiate between hvKp and cKp strains [43]. Therefore, the effect if any, of this deletion on the hypervirulent phenotype is unclear. In our study, of the 79 strains that were designated as hvKp based on having some combination of *iucA, rmpA, rmpA2, iroB*, and *peg-344*, 24/79 (30.4%) possessed all 5 biomarkers and 11/79 (13.9%) possessed *iucA* and *rmpA2*. Whether this later group or the remaining 44/79 (55.7%) strains that have an incomplete virulence marker profile are as virulent as hvKp strains that possess the complete repertoire is unclear. Studies are underway to resolve this issue.

Data support that the pVir-CR-hvKp4-like and pLVPK-like plasmids have both been acquired by ST11 Kp [16,26,27] in multiple independent events. Notably, the complete virulence plasmid appears to be more common among the ST11 sublineage expressing the K64 capsule type whereas the deletion variant appears to be more common in the sublineage expressing the K47 capsule type. Interestingly, ST11 strains possessing the K64 capsule type may be more virulent; however, both of the two sublineages are disseminating in China [26,28,29].

The main limitation in our study is that it was a retrospective study conducted at two hospitals, both located in Beijing. Furthermore, only a few the community-acquired Kp infection cases were identified; therefore, comparative analyses with this group may suffer from statistical bias. Thus, a future, large, international collaboration on hvKp is desirable. Lastly, as discussed, validation of strains designated as hvKp, especially those with an incomplete biomarker profile, in an outbred mouse model and measurement of total siderophore production, which has also shown to be highly predictive of the hvKp hypervirulent phenotype, would be of interest. Whole genomic sequencing of selected strains would also be insightful.

In conclusion, the prevalence of hvKp as defined by recently validated biomarkers was high in Beijing. HvKp may be replacing cKp as the dominant nosocomial pathotype. Unfortunately, detecting these infections in the absence of routine testing for hvKp by the clinical microbiology laboratory will be difficult. Therefore, it is critical that the clinician be aware of fact that hvKp causes infection not only in the community setting, but also in the healthcare setting. This is important since these infections have some specific management considerations compared to cKp. These include consideration of possible unrecognized sites of infection, which often manifest as occult abscesses and may require drainage, perhaps extended antimicrobial therapy, and, site-directed treatment (e.g. with meningitis, brain or prostatic abscesses, or endophthalmitis [vitrectomy, intra-vitreal antibiotics]) [4]. Ongoing surveillance of this new breed of MDR/XDR hvKp is critical.

## Supplementary Material

Supplemental Material

Supplemental Material
