# Supplementary material for: Hypervirulent Klebsiella pneumoniae is emerging as an increasingly prevalent K. pneumoniae pathotype responsible for nosocomial and healthcare-associated infections in Beijing, China
Source: Virulence. 2020 Sep 12;11(1):1215–24. doi: 10.1080/21505594.2020.1809322 (PMC7549996; doi:10.1080/21505594.2020.1809322)
Supplement: Supplemental Material [file KVIR_A_1809322_SM9151.doc]

Supplementary Table 1 Primers

| Items | Sequence |
| --- | --- |
| *pRmpA* |  |
| Forward | 5-ACTGGGCTACCTCTGCTTCA-3 |
| Reverse | 5-CTTGCATGAGCCATCTTTCA-3 |
| *pRmpA2* |  |
| Forward | 5-CTTTATGTGCAATAAG-GATGTT-3 |
| Reverse | 5-CCTCCTGGAGAGTAAGCATT-3 |
| *cRmpA* |  |
| Forward | 5-GTAATAGAGATATAAATATCATATTGA-3 |
| Reverse | 5-GCAATGGCCATTTGCGTTAG-3 |
| *iroB* |  |
| Forward  Reverse | 5-ATCTCATCATCTACCCTCCGCTC-3  5-GGTTCGCCGTCGTTTTCAA-3 |
| *iucA* |  |
| Forward | 5-AATCAATGGCTATTCCCGCTG-3 |
| Reverse | 5-CGCTTCACTTCTTTCACTGACAGG-3 |
| *peg344* |  |
| Forward | 5-CTTGAAACAGACCCTCCAGTC-3 |
| Reverse | 5-CCAGCGAATATAAATAACCCC-3 |
| *peg589* |  |
| Forward | 5-TGAACCCCATGAGGTCTATC-3 |
| Reverse | 5-GTGATGAATACCATACTGCGGC-3 |
| *K1* |  |
| Forward | 5-GTAGGTATTGCAAGCCATGC-3 |
| Reverse | 5-GCCCAGGTTAATGAATCCGT-3 |
| *K2* |  |
| Forward | 5-GGAGCCATTTGAATTCGGTG-3 |
| Reverse | 5-TCCCTAGCACTGGCTTAAGT-3 |
| *K5* |  |
| Forward | 5-GCCACCTCTAAGCATATAGC-3 |
| Reverse | 5-CGCACCAGTAATTCCAACAG-3 |
| *K20* |  |
| Forward | 5-CCGATTCGGTCAACTAGCTT-3 |
| Reverse | 5-GCACCTCTATGAACTTTCAG-3 |
| *K54* |  |
| Forward | 5-CATTAGCTCAGTGGTTGGCT-3 |
| Reverse | 5-GCTTGACAAACACCATAGCAG-3 |
| *K57* |  |
| Forward | 5-CGACAAATCTCTCCTGACGA-3 |
| Reverse | 5-CGCGACAAACATAACACTCG-3 |
| *rpoB* |  |
| Forward | 5-GGCGAAATGGCWGAGAACCA-3 |
| Reverse | 5-GAGTCTTCGAAGTTGTAACC-3 |
| *gapA* |  |
| Forward | 5-TGAAATATGACTCCACTCACGG-3 |
| Reverse | 5-CTTCAGAAGCGGCTTTGATGGCTT-3 |
| *mdh* |  |
| Forward | 5-TGAAATATGACTCCACTCACGG-3 |
| Reverse | 5-CTTCAGAAGCGGCTTTGATGGCTT-3 |
| *pgi* |  |
| Forward | 5-GAGAAAAACCTGCCTGTACTGCTGGC-3 |
| Reverse | 5-CGCGCCACGCTTTATAGCGGTTAAT-3 |
| *phoE* |  |
| Forward | 5-ACCTACCGCAACACCGACTTCTTCGG-3 |
| Reverse | 5-TGATCAGAACTGGTAGGTGAT-3 |
| *infB* |  |
| Forward | 5-CTCGCTGCTGGACTATATTCG-3 |
| Reverse | 5-CGCTTTCAGCTCAAGAACTTC-3 |
| *tonB* |  |
| Forward | 5-CTTTATACCTCGGTACATCAGGTT-3 |
| Reverse | 5-ATTCGCCGGCTGRGCRGAGAG-3 |
